# Assessing the relationship between systemic immune-inflammation index and mortality in patients with hypertrophic cardiomyopathy

**DOI:** 10.48101/ujms.v126.8124

**Published:** 2021-12-03

**Authors:** Ziqiong Wang, Haiyan Ruan, Liying Li, Xin Wei, Ye Zhu, Jiafu Wei, Xiaoping Chen, Sen He

**Affiliations:** a Department of Cardiology, West China Hospital of Sichuan University, Chengdu, China; b Department of Cardiology, Traditional Chinese Medicine Hospital of Shuangliu District, Chengdu, China; c Department of Cardiology and National Clinical Research Center for Geriatrics, West China Hospital of Sichuan University, Chengdu, China

**Keywords:** Systemic immune-inflammation index, all-cause mortality, hypertrophic cardiomyopathy, inflammation, risk stratification

## Abstract

**Background:**

This study investigates the predictive value of the systemic immune-inflammation index (SII), which was calculated as platelet × neutrophil/lymphocyte ratio, for all-cause mortality in patients with hypertrophic cardiomyopathy (HCM).

**Methods:**

A total of 360 HCM patients were enrolled. They were divided into three groups based on the tertiles of baseline SII. The association between SII and all-cause mortality was analyzed.

**Results:**

There were 53 HCM patients who died during a mean follow-up time of 4.8 years (min: 6 days and max: 10.8 years), and the mortality rate was 3.0 per 100 person years. The cumulative mortality rate was significantly different among the three tertiles of SII (*P* = 0.004), and the mortality rate in tertile 3 was much higher than that in the first two tertiles. In reference to tertile 1, the fully adjusted hazard ratios of all-cause mortality were 1.02 for the tertile 2 (95% confidence interval [CI]: 0.45–2.31, *P* = 0.966) and 2.31 for tertile 3 (95% CI: 1.10–4.87, *P* = 0.027). No significant interactions between SII and other variables were observed during subgroup analysis. The discriminative power was better for mid-term outcome than that for short-term or long-term outcomes. Sensitivity analyses including patients with normal platelet and white blood cell count have revealed similar results.

**Conclusion:**

SII was a significant risk factor for all-cause mortality in HCM patients. However, the discriminative power was poor to moderate. It could be used in combination with other risk factors in mortality risk stratification in HCM.

## Introduction

Hypertrophic cardiomyopathy (HCM) is the most frequent genetically transmitted heart disease with an estimated prevalence of 1:500 to 1:200 (1). HCM is characterized with a wide range of clinical features, which ranged from completely asymptomatic and normal lifespan to deleterious arrythmia, sudden cardiac death (SCD), severe thromboembolism, and end-stage heart failure (HF), resulting in HCM-related premature death (2–3). Although the overall prognosis is relatively good when managed in line with current clinical practice guidelines (4–5), excess mortality for HCM patients was still observed in different studies (6–8). Therefore, the desire to better risk stratify patients who were at high risk of an adverse outcome is an essential component in disease management.

Previous studies have revealed the existence of both local and systemic inflammation in HCM patients (7–9). Also, several studies indicated that markers of inflammation predicted adverse outcomes in HCM, such as high-sensitivity C reactive protein (CRP) (7), monocyte to high density lipoprotein cholesterol ratio (M/HDL-C) (8), and neutrophil to lymphocyte ratio (NLR) (10). A novel immune and inflammation index, namely, systemic immune-inflammation index (SII), which is calculated from platelet, neutrophil, and lymphocyte counts, has been examined as a prognostic factor for clinical outcomes in cancer patients (11–12) and in patients with cardiovascular diseases, such as coronary artery disease (13–14), hypertension (15), pulmonary embolism (16), and acute ischemic stroke (17). However, to date, the predictive ability of SII for mortality has not been reported in HCM. The present study investigated the prognostic value of SII for mortality in HCM patients from a tertiary referral center.

## Methods

### Study patients

**Figure 1:**
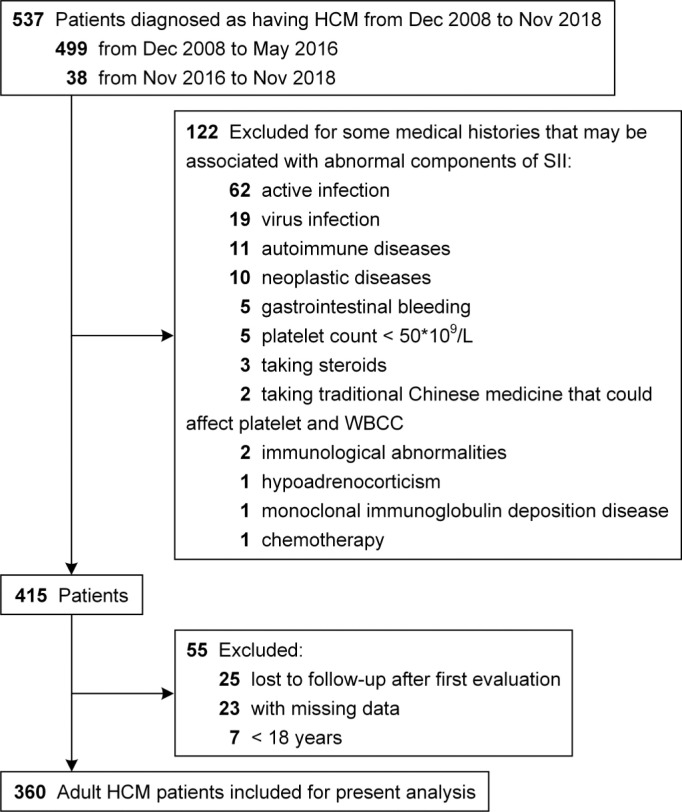
Study flow diagram.

From December 2008 to November 2018, 537 patients with a diagnosis of HCM were consecutively enrolled at West China Hospital of Sichuan University, Chengdu, China. All patients underwent 2D transthoracic echocardiography examinations by standard techniques, and the diagnosis of HCM was based on the presence of increased left ventricular (LV) wall thickness (≥15 mm) that was not solely explained by abnormal loading conditions (18). Based on the inclusion and exclusion criteria (Figure 1), a total of 360 adult HCM patients were finally included.

This study was conducted according to the principles of the Declaration of Helsinki and was approved by the Biomedical Research Ethics Committee, West China Hospital of Sichuan University (approval number: 2019-1147). This study has been registered on the website of Chinese Clinical Trial Registry (http://www.chictr.org.cn/showproj.aspx?proj=48695). An informed consent was waived due to the retrospective nature of the study. Other detailed information has been reported in three recently published studies (19–21).

### Clinical evaluations

Baseline characteristics were collected from medical records by experienced physicians, and these characteristics mainly included medical history, therapy, 12-lead echocardiogram, Doppler echocardiography, and peripheral blood parameters. A twice-entry method was used for data entry. When values of the two entries were consistent, they would enter the database; otherwise, the raw data would be checked.

Platelet, neutrophil, and lymphocyte were tested using a Sysmex XN-9000 analyzer (Sysmex Corporation, Kobe, Japan), and the values were collected at the time of hospital admission. In this system, normal ranges of platelet count, white blood cell count (WBCC), neutrophil count, and lymphocyte count are 100–300*10^9^/L, 4–10*10^9^/L, 1.8–6.3*10^9^/L, and 1.1–3.2*10^9^/L, respectively. SII was calculated as total peripheral platelets count (P) × neutrophil-to-lymphocyte ratio (N/L) (SII = P × N/L ratio) (22).

### Study endpoints

The endpoint was all-cause mortality, which included HCM-related death, other cardiac death (e.g. myocardial infarction), non-cardiac death, and unexplained death. Specifically, HCM-related death was defined as a composite of HF-related death, stroke-related death (SCD), and other specific HCM-related death. Follow-up was carried out via medical records or telephone contact with the patients themselves and/or referring relatives. All patients were followed from the first evaluation up to the endpoint or the most recent evaluation.

### Statistical analysis

To quantify, in a simple form, the relationship between SII and all-cause mortality, the patients were divided into three groups according to baseline SII, which were categorized separately as follows: tertile 1 (<246.04×10^9^/L), tertile 2 (246.04–424.90×10^9^/L), and tertile 3 (≥424.91×10^9^/L). For continuous variables, data were presented as median with interquartile range (IQR) since all variables were skewed distribution based on Shapiro–Wilk test. For categorical variables, data were presented as number (percentage). Kruskal–Wallis test, Chi-square test, or Fisher exact test were used, as appropriate, to compare baseline characteristics among the three groups.

The Kaplan–Meier method was used to evaluate the association between SII and all-cause mortality, and log-rank tests were used for comparisons. Several Cox proportional hazard regression models were constructed to assess the prognostic value. Variables for inclusion were carefully chosen to ensure parsimony of multivariate models. Two multivariate models were constructed. Model 1, the basic model, adjusted for demographic data, including age and gender. Model 2, the final model, baseline variables that were considered to be related to the study endpoint based on previous meta-analysis were pre-specifed, which included NYHA function class, syncope/presyncope, maximal LV wall thickness (MWT), and resting LV outflow track obstruction (LVOTO) (23), and were foreced into the final model. Besides, variables that showed a significant relationship with mortality in univariate analysis (*P* < 0.05) were entered into the multivariate model as well and then were sought using a backward stepwise modeling approach (*P* = 0.05 for inclusion and *P* = 0.10 for exclusion) to include some of them in the final model. Additionally, stratified analysis was condcuted to assess the consistency of association between SII and all-cause mortality in various subgroups, and their interaction effect was tested. Meanwhile, the robustness of the main results was assessed by calculation of E-values. E-values could assess the potential for unmeasured confounding between SII and all-cause mortality, and it quantifies the required magnitude of an unmeasured confounder that could negate the observed association between SII and all-cause mortality (24).

Furthermore, a time-dependent receiver operating characteristic curve was generated to evaluate the discriminative power of SII in predicting all-cause mortality over time. Finally, we assessed the relation between SII and all-cause mortality in patients with normal platelet and WBCC as a sensitivity analysis in case some other unknown reasons affecting the components of SII had not been ruled out.

The statistical analyses were performed with the use of R software, version 4.1.0 (R Project for Statistical Computing). For all statistical analyses, a two-sided *P*-value of 0.050 was considered statistically significant.

## Results

### Baseline characteristics

The present study comprised 360 patients (male, 53.89%) with a median age of 56.00 (IQR, 44.00–66.00) years, and SII ranged from 6.55 to 2,856.84×10^9^/L (median, 336.95×10^9^/L; IQR, 216.57–502.28×10^9^/L). According to the tertiles of baseline SII, there were 119 patients in the lowest tertile, 118 in the second tertile, and 123 in the highest tertile. The levels of platelet count, WBCC, neutrophil count, and monocyte count significantly increased across the SII tertiles. The prevalence of hypertension and the use of aspirin significantly also increased across the SII tertiles. These patients with higher SII had significantly lower levels of hemoglobin and lymphocyte count, as well as lower prevalence of palpitation. Other detailed data about baseline characteristics are presented in Table 1.

**Table 1:** Baseline characteristics.

Variables	All (*n* = 360)	SII, tertile 1 (*n* = 119)	SII, tertile 2 (*n* = 118)	SII, tertile 3 (*n* = 123)	*P*
Gender: male	194 (53.89%)	71 (59.66%)	58 (49.15%)	65 (52.85%)	0.257
Age (years)	56.00 [44.00, 66.00]	56.00 [44.00, 64.50]	55.00 [44.25, 69.00]	56.00 [45.00, 66.00]	0.844
Family history of HCM	32 (8.89%)	12 (10.08%)	12 (10.17%)	8 (6.50%)	0.519
Family history of SCD	14 (3.89%)	2 (1.68%)	4 (3.39%)	8 (6.50%)	0.147
MYHA III-IV	59 (16.39%)	18 (15.13%)	15 (12.71%)	26 (21.14%)	0.189
Symptoms					
Dyspnea	204 (56.67%)	71 (59.66%)	60 (50.85%)	73 (59.35%)	0.298
Chest pain	209 (58.06%)	72 (60.50%)	72 (61.02%)	65 (52.85%)	0.352
Syncope/presyncope	120 (33.33%)	42 (35.29%)	48 (40.68%)	30 (24.39%)	0.024
Palpitation	143 (39.72%)	62 (52.10%)	44 (37.29%)	37 (30.08%)	0.002
Medical history					
Prior TE	15 (4.17%)	2 (1.68%)	5 (4.24%)	8 (6.50%)	0.159
Vascular disease	29 (8.06%)	9 (7.56%)	10 (8.47%)	10 (8.13%)	0.967
Hypertension	116 (32.22%)	26 (21.85%)	43 (36.44%)	47 (38.21%)	0.012
Diabetes mellitus	29 (8.06%)	6 (5.04%)	9 (7.63%)	14 (11.38%)	0.190
Atrial fibrillation	62 (17.22%)	22 (18.49%)	21 (17.80%)	19 (15.45%)	0.805
Therapy					
Aspirin	72 (20.00%)	11 (9.24%)	27 (22.88%)	34 (27.64%)	0.001
Clopidogrel	21 (5.83%)	8 (6.72%)	4 (3.39%)	9 (7.32%)	0.378
Warfarin	38 (10.56%)	15 (12.61%)	10 (8.47%)	13 (10.57%)	0.585
Statin	110 (30.56%)	30 (25.21%)	41 (34.75%)	39 (31.71%)	0.265
Beta blocker	274 (76.11%)	92 (77.31%)	99 (83.90%)	83 (67.48%)	0.011
ACEI/ARB	72 (020.00%)	24 (20.17%)	23 (19.49%)	25 (20.33%)	0.985
Intervention of obstruction					0.519
None	313 (86.94%)	100 (84.03%)	101 (85.59%)	112 (91.06%)	
Alcohol septal ablation	41 (11.39%)	16 (13.45%)	15 (12.71%)	10 (8.13%)	
Septal myectomy	6 (1.67%)	3 (2.52%)	2 (1.69%)	1 (0.81%)	
Devices					0.460
None	320 (88.89%)	101 (84.87%)	108 (91.53%)	111 (90.24%)	
Pacemaker	14 (3.89%)	5 (4.20%)	4 (3.39%)	5 (4.07%)	
ICD	26 (7.22%)	13 (10.92%)	6 (5.08%)	7 (5.69%)	
Blood index					
Hgb (g/L)	139.00 [128.00, 151.00]	141.00 [129.00, 152.00]	140.50 [132.00, 151.75]	134.00 [120.00, 148.00]	0.005
Platelet count (109/L)	146.00 [112.00, 185.00]	112.00 [88.50, 135.00]	153.50 [124.00, 185.50]	177.00 [148.50, 224.50]	<0.001
WBCC (10^9^/L)	6.26 [5.15, 7.44]	5.44 [4.64, 6.37]	6.31 [5.22, 7.24]	7.37 [5.98, 8.66]	<0.001
Lymphocyte count (10^9^/L)	1.69 [1.36, 2.08]	1.85 [1.52, 2.26]	1.81 [1.47, 2.17]	1.42 [1.07, 1.72]	<0.001
Neutrophil count (10^9^/L)	3.78 [3.05, 4.90]	2.93 [2.44, 3.51]	3.77 [3.21, 4.60]	5.22 [4.03, 6.58]	<0.001
Monocyte count (10^9^/L)	0.35 [0.28, 0.46]	0.32 [0.26, 0.39]	0.35 [0.27, 0.46]	0.41 [0.30, 0.53]	<0.001
Creatinine (μmol/L)	80.00 [67.00, 92.65]	80.00 [66.20, 87.85]	80.00 [67.32, 90.92]	80.40 [68.00, 95.00]	0.454
Uric acid (μmol/L)	362.50 [299.08, 433.48]	357.00 [301.75, 421.50]	366.50 [308.25, 433.50]	367.10 [291.20, 446.15]	0.723
TG (mmol/L)	1.28 [0.97, 1.96]	1.20 [0.96, 1.87]	1.46 [1.01, 2.16]	1.24 [0.90, 1.72]	0.087
HDL-C (mmol/L)	1.27 [1.04, 1.54]	1.30 [1.11, 1.54]	1.23 [1.01, 1.54]	1.28 [1.02, 1.52]	0.339
LDL-C (mmol/L)	2.44 [1.87, 2.94]	2.56 [1.81, 3.07]	2.37 [1.95, 2.85]	2.37 [1.83, 2.92]	0.395
Echocardiographic parameters					
LVEDD (mm)	43.00 [40.00, 47.00]	43.00 [39.50, 46.00]	43.00 [40.00, 47.75]	43.00 [39.00, 47.50]	0.566
LA diameter (mm)	40.00 [36.00, 45.00]	40.00 [36.00, 45.00]	40.00 [36.00, 45.00]	40.00 [35.00, 45.00]	0.800
MWT (mm)	19.00 [17.00, 22.00]	19.00 [17.00, 22.00]	19.00 [16.00, 22.00]	19.00 [16.50, 22.00]	0.540
LVEF (%)	69.00 [64.00, 73.00]	69.00 [64.50, 73.50]	68.00 [64.00, 71.00]	69.00 [63.00, 73.00]	0.117
Resting LVOTG ≥30 mm Hg	156 (43.33%)	54 (45.38%)	55 (46.61%)	47 (38.21%)	0.362

Values are median (IQR) or n (%).

SII: systemic inflammatory-immune index; HCM: hypertrophic cardiomyopathy; SCD: sudden cardiac death; NYHA: New York Heart Association; TE: thrombo-embolic event; ACE: angiotensin-converting enzyme; ARB: angiotensin receptor blocker; ICD: implantable cardioverter defibrillator; Hgb: hemoglobin; WBCC: white blood cell count; TG: triglyceride; HDL-C: high-density lipoprotein cholesterol; LDL-C: low-density lipoprotein cholesterol; LVEDD: left ventricular end-diastolic dimension; LA: left atrial; MWT: maximal left ventricular wall thickness; LVEF: left ventricular ejection fraction; LVOTG: left ventricular outflow tract gradient.

### Correlation

SII negatively correlated with hemoglobin (*r* = −0.23, *P* < 0.001) and lymphocyte count (*r* = −0.29, *P* < 0.001) and positively correlated with platelet count (*r* = 0.44, *P* < 0.001), WBCC (*r* = 0.57, *P* < 0.001), neutrophil count (*r* = 0.73, *P* < 0.001), and monocyte count (*r* = 0.26, *P* < 0.001). These correlations were somewhat weak to moderate. Besides, SII showed no correlation with creatinine, uric acid, blood lipid parameters, and echo data (Supplementary Table 1).

### Study endpoints

During a follow-up period of 1,744.0 person-years (PYs) (median, 4.8 years; IQR, 2.8–6.8 years), there were 53 (14.7%) all-cause mortalities with a mortality rate of 3.0 (95% confidence interval [CI]: 2.2–3.8) per 100 PYs.

The specific causes of deaths were as follows: 14 HF-related deaths, 11 stroke-related deaths, 8 SCDs, 2 HCM-related postoperative deaths, 1 other cardiac death, and 12 non-cardiac deaths. The cause of death could not be determined in five patients.

### Association between SII and mortality

**Figure 2:**
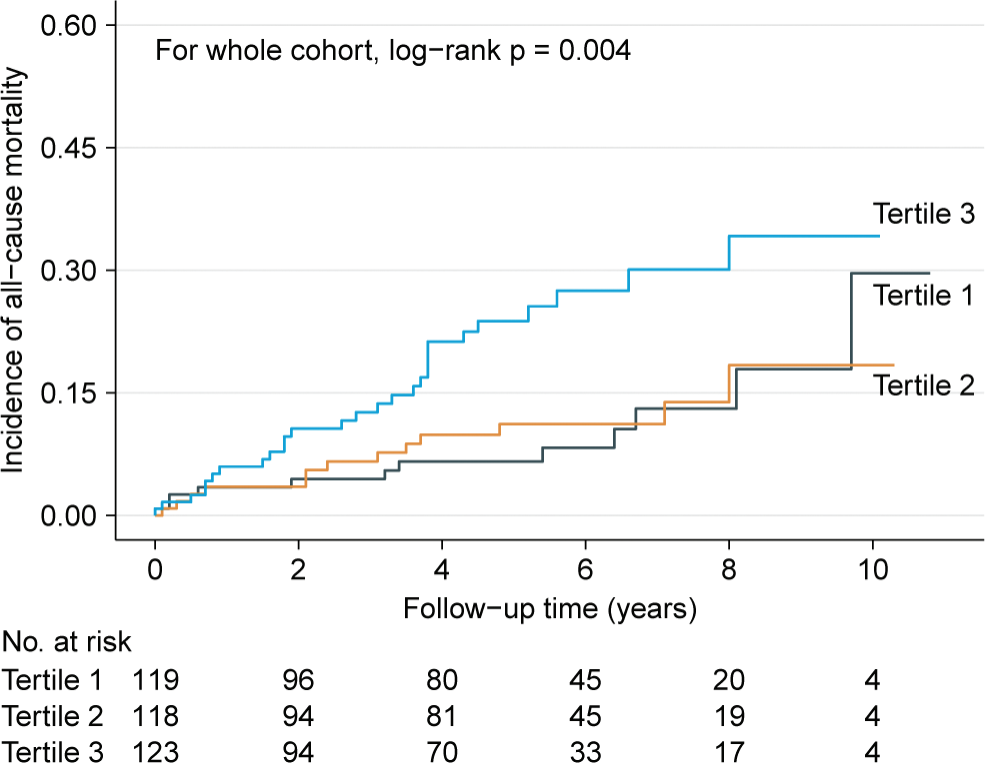
Kaplan–Meier analysis showing cumulative all-cause mortality by baseline SII tertiles.

**Figure 3:**
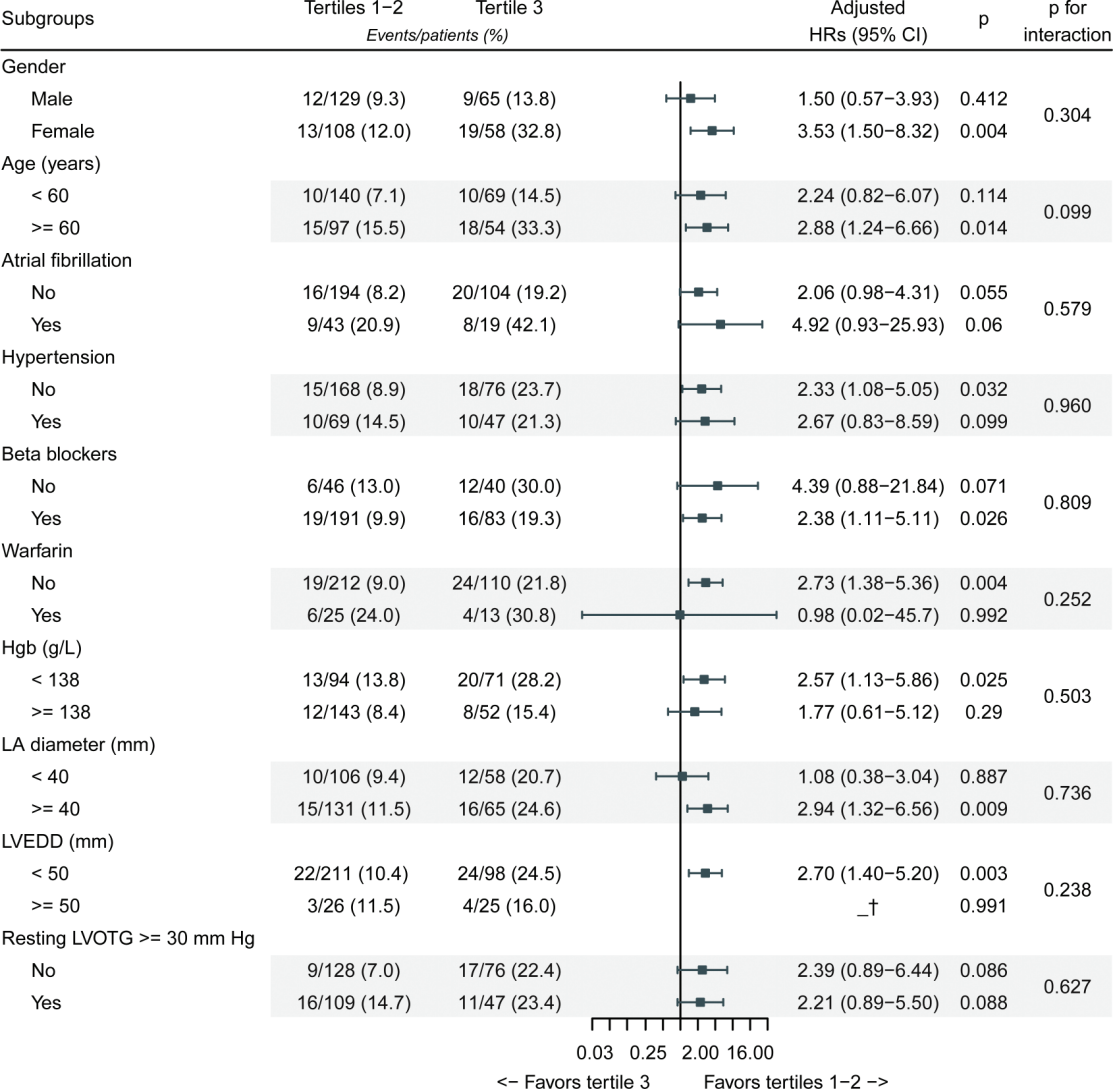
Stratified analyses of all-cause mortality. *Note*: Each stratification adjusted for age, gender, dyspnea, uric acid, TG, monocyte count, LVEDD, LA diameter, and LVEF, except the stratification factor itself. The *P*-value for interaction represents the likelihood of interaction between variable and SII tertiles. Abbreviations as in Tables 1 and 2. ^†^Adjusted HR = 0.

The all-cause mortality rates were 2.0 (95% CI: 0.9–3.1), 2.2 (95% CI: 1.0–3.3), and 5.1 (95% CI: 3.3–7.0) per 100 PYs in the tertile 1, tertile 2, and tertile 3, respectively (Table 2). The cumulative mortality rate was significantly different among the three tertiles of SII (log-rank *P* = 0.004, Figure 2), and the mortality rate in tertile 3 was much higher than that in the first two tertiles. Univariate Cox regression analysis indicated that SII was a significant risk factor for future all-cause mortality (Table 2). Other variables that could predict all-cause mortality have been shown in Supplementary Table 2. In reference to tertile 1, fully adjusted HRs were 1.02 for tertile 2 (95% CI: 0.45–2.31, *P* = 0.966) and 2.31 for tertile 3 (95% CI: 1.10–4.87, *P* = 0.027) (Table 2). Due to the similar mortality rates in the first two tertiles, we combined them into one to perform the stratified analysis (tertiles 1–2 vs. tertile 3). It was found that the mortality risk was consistently higher in tertile 3 than in tertiles 1–2 in all subgroups. No interaction effect was observed between SII and other variables for mortality prediction (Figure 3), which suggested that SII was an independent predictor for all-cause mortality in HCM patients, and other variables were not confounders or effect modifier.

**Table 2:** Associations of SII with all-cause mortality.

	SII Tertile 1	SII Tertile 2	SII Tertile 3
No. of patients (*n*)	119	118	123
Endpoints (*n*)	12	13	28
Follow-up (PYs)	598.7	597.7	547.6
Mortality rates (95% CI)*	2 [0.9, 3.1]	2.2 [1, 3.3]	5.1 [3.3, 7]
Unadjusted HRs (95% CI), *P*	1	1.09 [0.50, 2.40], 0.822	2.53 [1.29, 4.98], 0.007
Adjusted HRs (95% CI), *P*			
Model 1	1	1.00 [0.45, 2.20], 0.998	2.52 [1.28, 4.96], 0.008
Model 2	1	1.02 [0.45, 2.31], 0.966	2.31 [1.10, 4.87], 0.027

Model 1 with adjustment for age and gender.

Model 2 with adjustment for age, gender, syncope/presyncope, dyspnea, NYHA III-IV, uric acid, TG, monocyte count, LA diameter, LVEDD, LVEF, MWT, and resting LVOTO.

PYs: person-years; CI: confidence interval; HRs: hazard ratios; other abbreviations as in Table 1. Per 100 PYs.

In addition, the E-value estimates for the effects of SII on all-cause mortality were 4.05 (lower limit of CI, 1.43) for tertile 3. This suggested that the main findings should be robust, unless an unmeasured confounder existed with a higher relative risk than the above-mentioned E-values.

### Discriminative power of SII for all-cause mortality

**Figure 4:**
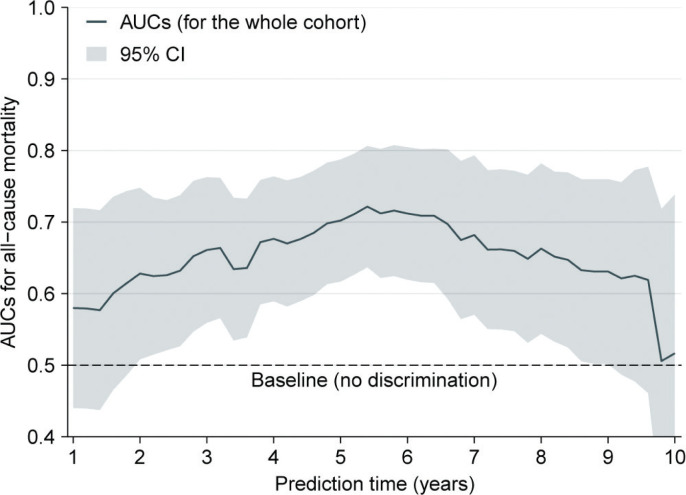
Time-dependent AUCs. Note: The curve was based on the AUCs, which was calculated every 0.2 years (from 1 to 10 years). In the figure, the solid line depicts the AUCs, and the ribbon represents 95% CI. Abbreviations as in Tables 1 and 2.

We assessed the discriminative power of SII for all-cause mortality at different timepoints. The time-dependent area under curve (AUC) at 1-year follow-up was 0.580. With time prolongation, AUC increased to a maximum of 0.722 at 5.4-year follow-up and then gradually decreased to 0.517 at 10-year follow-up, indicating a dynamic change of the discriminative ability (Figure 4).

### Sensitivity analysis

Sensitivity analysis including only patients with normal platelet and WBCC (*n* = 269, all-cause mortality = 39) revealed similar results with the main analyses. Kaplan–Meier analysis demonstrated significantly higher incidence of all-cause mortality across the three tertiles (log-rank *P* = 0.018, Supplementary Figure 1). With tertile 1 as reference, unadjusted HRs were 1.38 for tertile 2 (95% CI: 0.54–3.51, *P* = 0.495) and 2.81 for tertile 3 (95% CI: 1.24–6.36, *P* = 0.013). Although the association between SII and all-cause mortality was reduced after adjustment for confounders, it did not change materially. Fully adjusted HRs were 1.27 for tertile 2 (95% CI: 0.45–3.55, *P* = 0.650) and 2.22 for tertile 3 (95% CI: 0.92–5.35, *P* = 0.074) when compared to tertile 1. Time-dependent AUCs showed similar findings with the main analyses (at 1-year: 0.525, at 5.4-year: 0.709, and at 10-year: 0.500) (Supplementary Figure 2).

## Discussion

In this retrospective cohort study of patients with HCM, we evaluated the ability of SII, a novel inflammatory biomarker, to predict the all-cause mortality. Higher SII was significantly associated with increased risk of all-cause mortality. The discriminative power in predicting mid-term outcome was better than that for short-term or long-term outcomes. Sensitivity analysis of patients with normal platelet and normal WBCC demonstrated similar results with the main analysis. Our study suggested that SII might be a potentially useful noninvasive predictor for mortality in HCM.

SII is determined by three components, namely, neutrophils, lymphocytes, and platelets, which reflect host inflammatory and immune status. It was initially proposed by Hu et al. as a powerful prognostic indicator for poor outcome in patients with hepatocellular carcinoma (22). After that, series of studies regarding the association between SII and clinical outcome in a variety of neoplastic diseases have been conducted, and all studies consistently confirmed the previous finding (11–12). Due to high levels of platelets and neutrophils while low level of lymphocytes, a higher SII indicates an elevated inflammatory and a suppressed immune response, both of which have played decisive roles in the pathogenesis, progression, and metastasis of neoplastic diseases (25, 26). From this point of view, increased SII is biologically plausible to associate with adverse prognosis for patients with neoplastic diseases.

It is widely acknowledged that the inflammation has played a central role in cardiovascular diseases, especially in coronary artery disease. Previous studies have shown that SII is a potential indicator for clinical endpoints for patients with coronary artery disease (13, 14). The same was found in patients with hypertension (15), acute ischemic stroke (17), and chronic HF (27). However, no previous study has discussed the predictive value of SII in HCM. In the present cohort study of HCM, we firstly proved its predictive value for all-cause mortality, which consisted of SCD, HF-related death, stroke-related death, cancer-related death, etc. The precise mechanisms of poor prognosis for some HCM patients have not been fully elucidated, but the chronic inflammation is possibly involved. Myocardial tissue changes, such as inflammatory cell infiltration and neutrophil extracellular traps formation due to myocyte disarray in the early stage, platelet activation, micro-vascular thrombosis and fibrosis pathway activation in the intermediate stage, myocardial fibrosis, and remodeling in the late phase, might be related to the phenotypic variability of HCM (28). Also, some systemic inflammatory biomarkers were also found to be significantly associated with adverse outcomes in HCM. In a retrospective study of 490 HCM patients, Zhu et al. demonstrated that patients with higher levels of plasma high-sensitivity C-reactive protein were at higher risk of all-cause mortality and cardiovascular death (7). In another study, Ekizler et al. showed that HCM patients with higher M/HDL-C had higher risk of malignant arrhythmic events and cardiovascular death (8). In a prospective observational study, Ozyilmaz et al. revealed that a higher NLR was associated with a higher 5-year risk of SCD in patents with HCM (10). In corroboration with that, our previous studies also found a relationship between NLR (29), red blood cell distribution width (30), and all-cause mortality, and the present study further extended the inflammatory biomarkers into SII. In addition, we observed that the discriminative power of SII for all-cause mortality was not stable at different timepoints, and it was better for mid-term outcome than that for short-term or long-term outcomes. Whether this dynamic change is a chance finding or not, it will require further studies. SII is a biomarker comprehensively reflecting the inflammatory and immune mechanism. However, the discriminative power was moderate at best, highlighting the difficulty of risk stratification in HCM patients if based on only a single risk factor due to the heterogeneity of HCM per se.

In the present study, the all-cause mortality rate was 14.7%, and the cardiovascular mortality rate was about 10.0%. In another HCM cohort from Fuwai Hospital, Bejing, China, the all-cause mortality and cardiovascular mortality rates were 7.8 and 6.1%, respectively (7). In a multiple European centers-based study, the authors focused on a composite study endpoint, which included SCD or equivalent end point, HF-related death or equivalent end point (heart transplant), other cardiovascular causes, and other unknown causes. The composite study endpoint occurred in 721 HCM patients with a prevalence of 14.7%, and the cardiovascular mortality rate was approximately 10.0% (6). In another study, based on two American centers, Marron et al. showed that the event rate for all-cause mortality and HCM-related mortality was 8.0 and 4.0%, respectively (5). For a Turkey HCM cohort, the all-cause mortality rate was 5.8%, and the cardiovascular death rate was 3.6%. However, there was a high prevalence (10.3%) of malignant arrhythmic events, including an occurrence of SCD, sustained ventricular tachycardia/ventricular fibrillation, or implantable cardioverter–defibrillator discharge in their study (8). Evidently, there are still discrepancies with regard to the poor outcomes across different studies. Although there are different baseline characteristics and ethnicities in these studies that might partially be the cause, the generally poor prognosis is still present.

Notably, most of the HCM patients in our study had well-preserved systolic function with the median value of LV ejection fraction (LVEF) at 69%. However, HCM is characterized by normal to hyperdynamic LVEF. Only a small portion of patients (4–9%) would develop systolic dysfunction with LVEF < 50% and progressed into end-stage HCM (31). In our study, the prevalence of end-stage HCM was 4.4%. There were some slight differences with regard to the values of echocardiographic parameters, including LV end-diastolic diameter, left atrial diameter, and MWT and LVEF between our study and previous studies (7, 8, 10), which might be caused by different ethnicities, different gender composition, and the variety of comorbidities. But, all supported the characteristics of non-dilated ventricles in HCM. Additionally, we did not find correlations between SII and those aforementioned echocardiographic diameters, and those data showed homogeneity across the three SII tertiles. SII represents a systemic inflammatory index. There is limited data about how SII would directly influence cardiac structure and cardiac function. A previous study, investigating the relationships between several inflammatory biomarkers and myocardial fibrosis, systolic and diastolic functions, and the degree of cardiac hypertrophy in HCM patients, also found no correlations between systemic inflammation and systolic function. However, only some circulating inflammatory markers were associated with myocardial fibrosis (e.g. interleukin-6, interleukin-4, and monocyte attractant protein-1), the degree of hypertrophy (e.g. fractalkine), or diastolic dysfunction (e.g. interferon-γ-inducible protein 10, interleukin-10, and transforming growth factor-β1) (32). Those inflammatory markers belong to cytokines/chemokines, which may mediate the pathological process of cardiac hypertrophy and fibrosis in HCM directly. Therefore, the correlation between the systemic inflammatory index derived from peripheral blood cells and the cardiac structure and cardiac function needs further studies.

The present study revealed the predictive value of SII for all-cause mortality in HCM patients. SII is a readily available inflammatory biomarker that could be easily obtained from complete blood cell test. Patients with higher SII might be given a closer clinical monitoring and more aggressive therapy. Our study, however, has certain limitations. First, the HCM patients enrolled in our study come from a single tertiary referral center, and the study was carried out retrospectively. Besides, the relatively small sample size might reduce the statistical power. Second, we focused on the all-cause mortality but not the HCM-related mortality due to the limited number of specific causes. The latter one might better reflect the clinical importance of SII in HCM, but we can still get some useful information. Third, although we have adjusted for certain potential confounding factors, some other well-established risk factors, such as non-sustained ventricular tachycardia and B-type natriuretic peptide, were not included due to incomplete data, which might reduce the strength of our findings. But, we have collected most of the variables as much as possible, which had showed significant predictive value for all-cause mortality and cardiovascular mortality in HCM patients in previous studies and included them in a multivariate model for adjustment. Fourth, our study only demonstrated a possible association of an inflammatory state and the clinical severity of HCM rather than a causal relationship between SII and the prognosis of HCM. Further large-scale studies are warranted to validate the present findings and clarify the prognostic utility of SII in HCM.

## Conclusion

Our study indicates that a higher SII is associated with increased risk for all-cause mortality in HCM patients, but the discriminative power is only poor to moderate. Further studies are needed to ascertain the predictive value of SII for adverse outcomes, especially for the specific HCM-related cardiovascular events. The index might be used in combination with other risk factors to improve the risk prediction of adverse outcomes for HCM patients.
